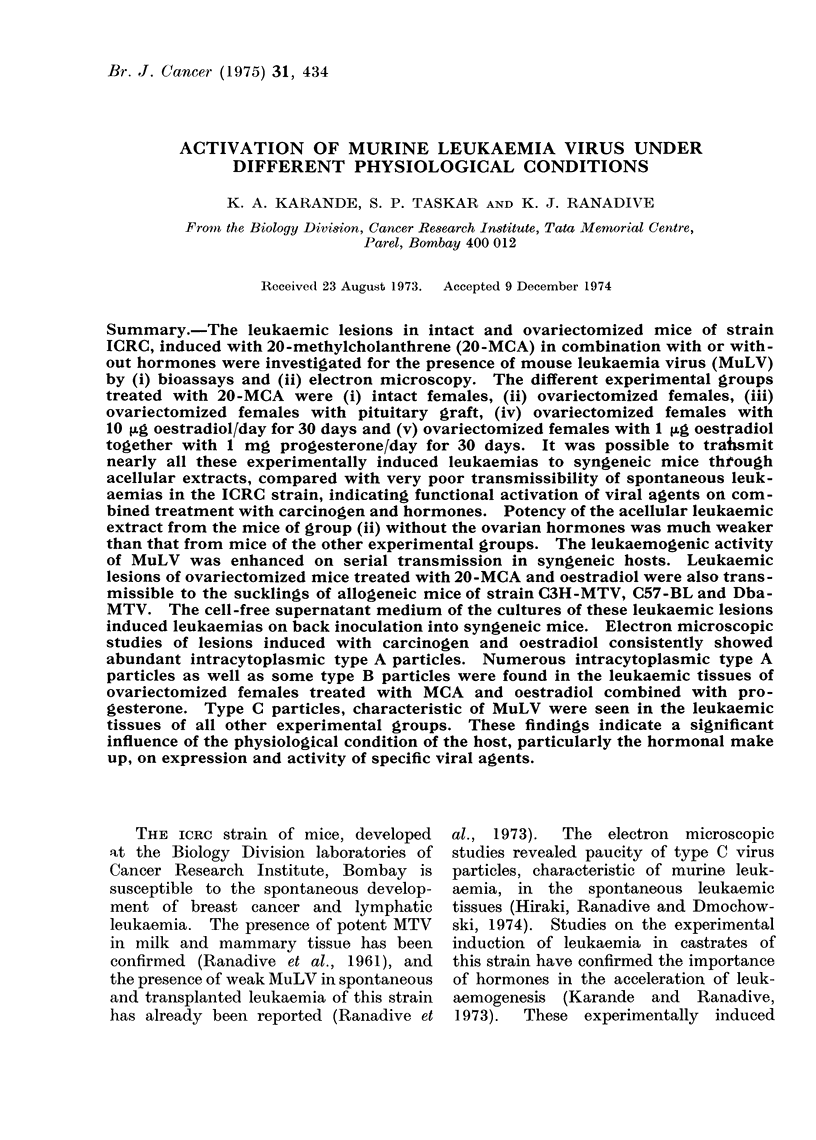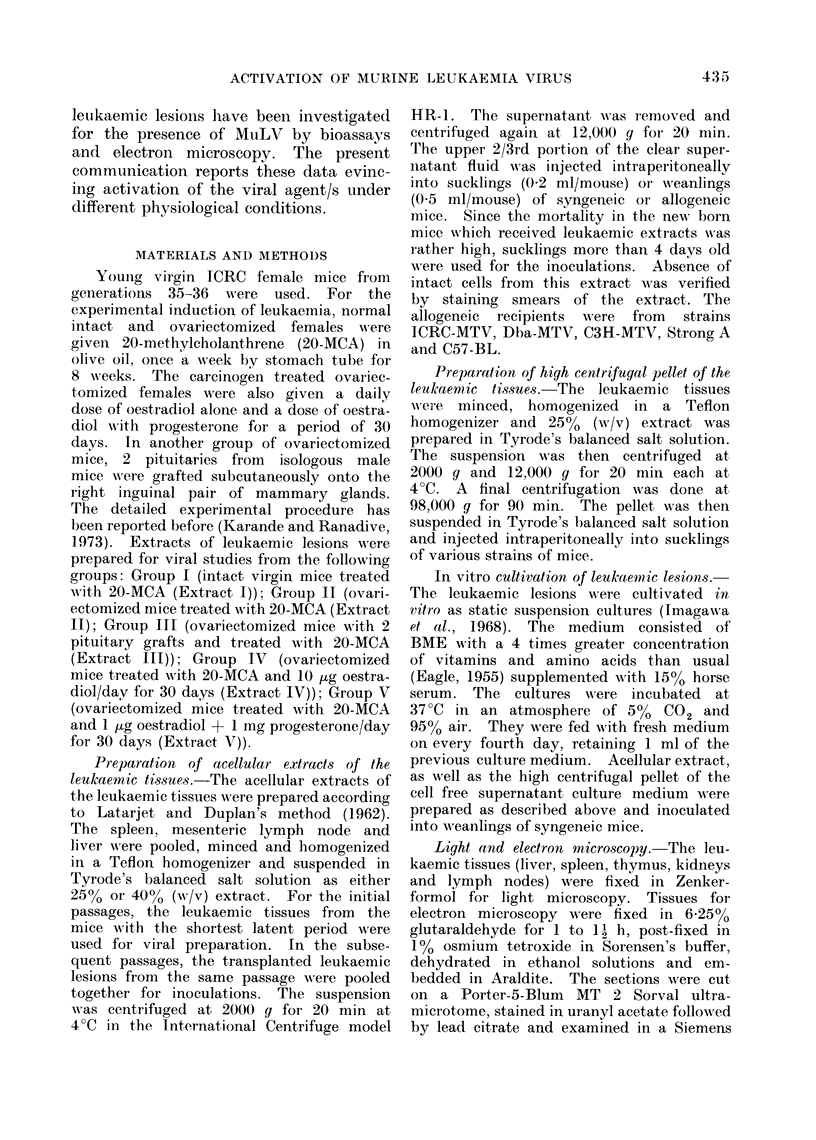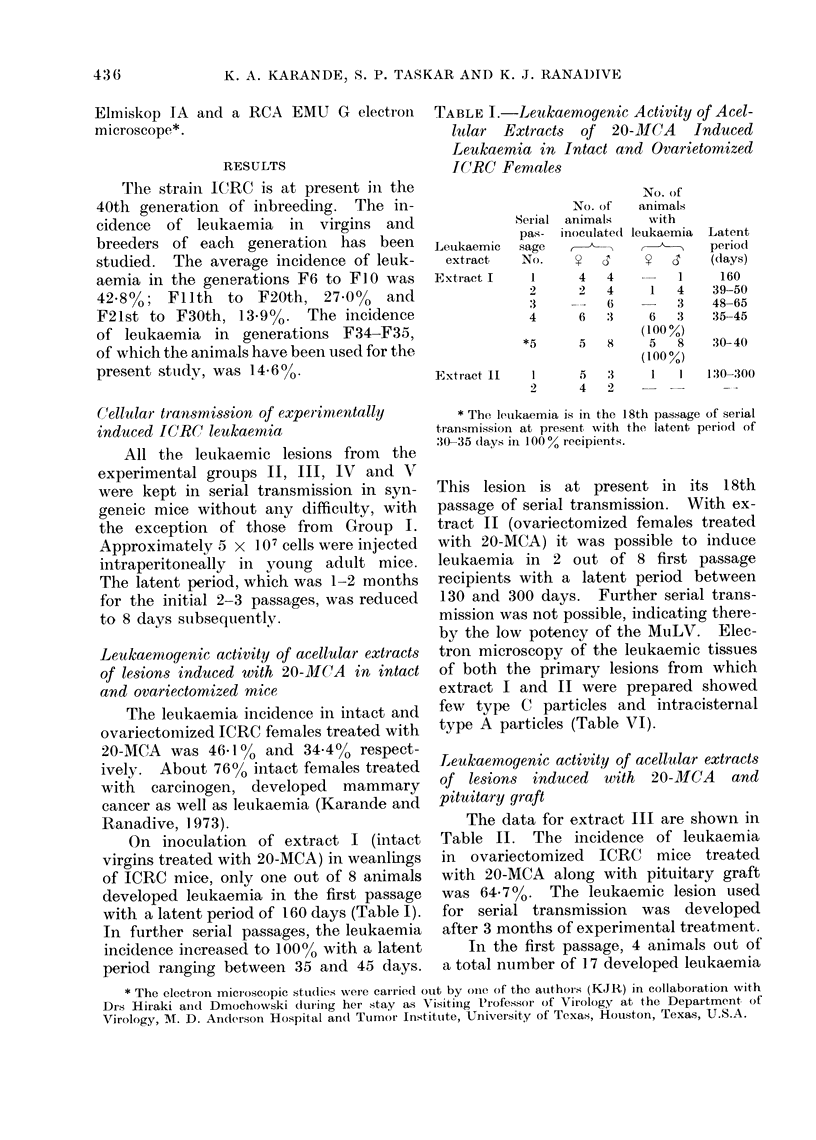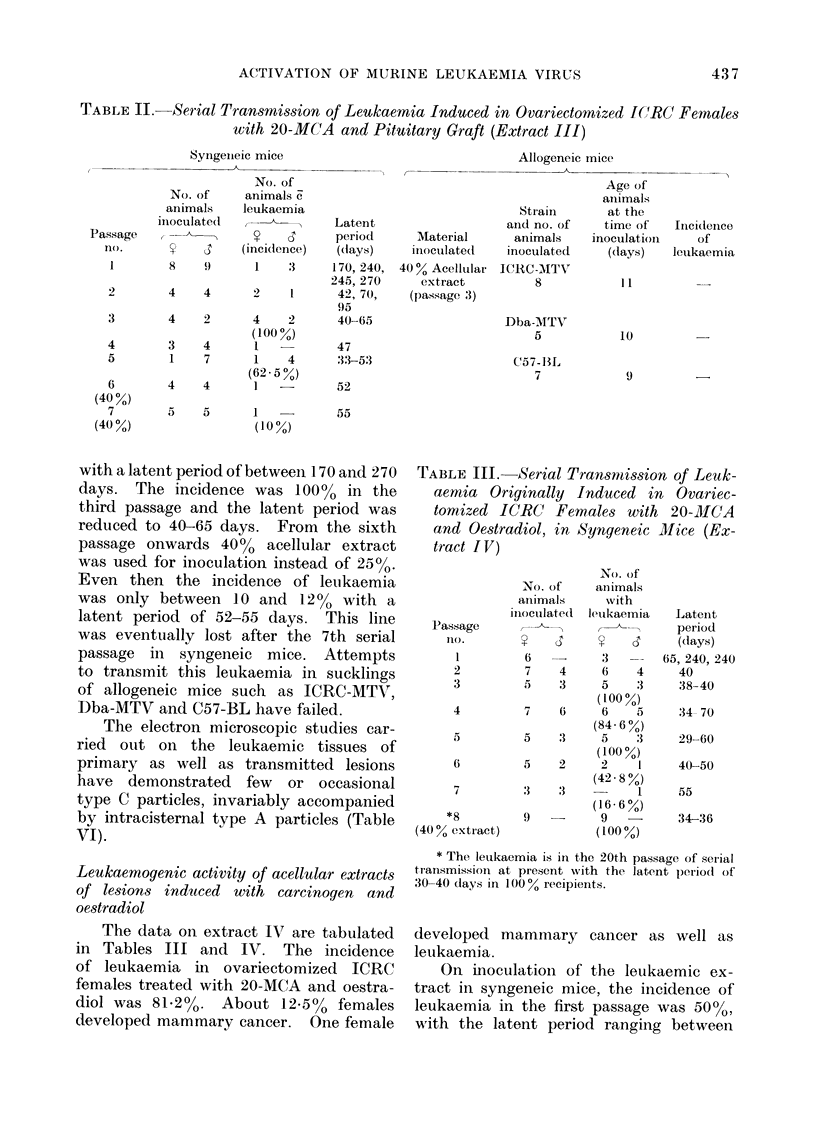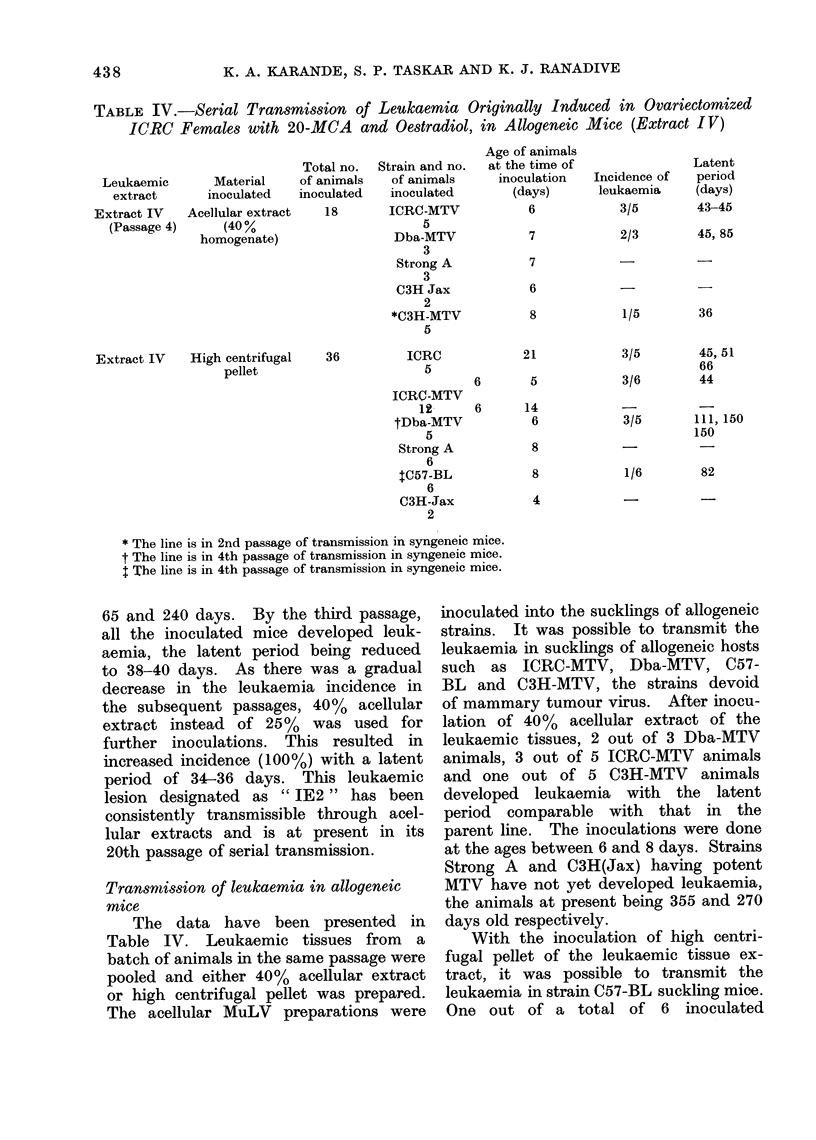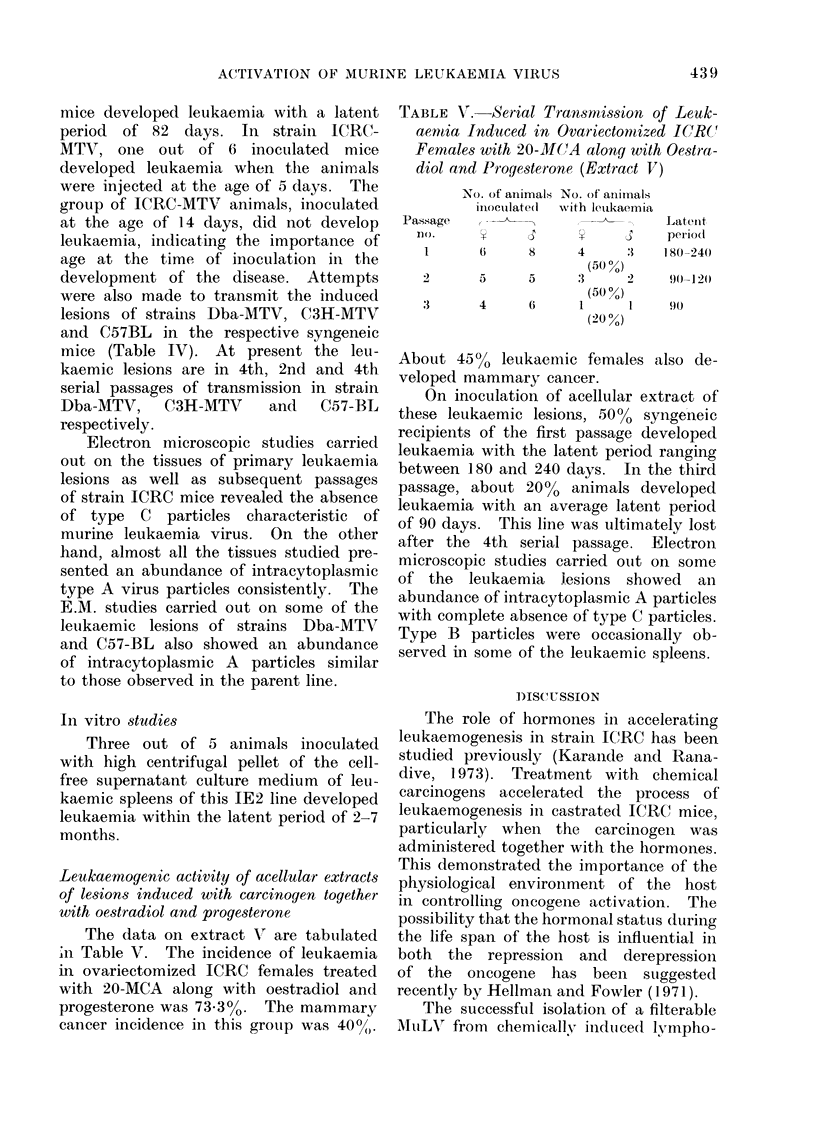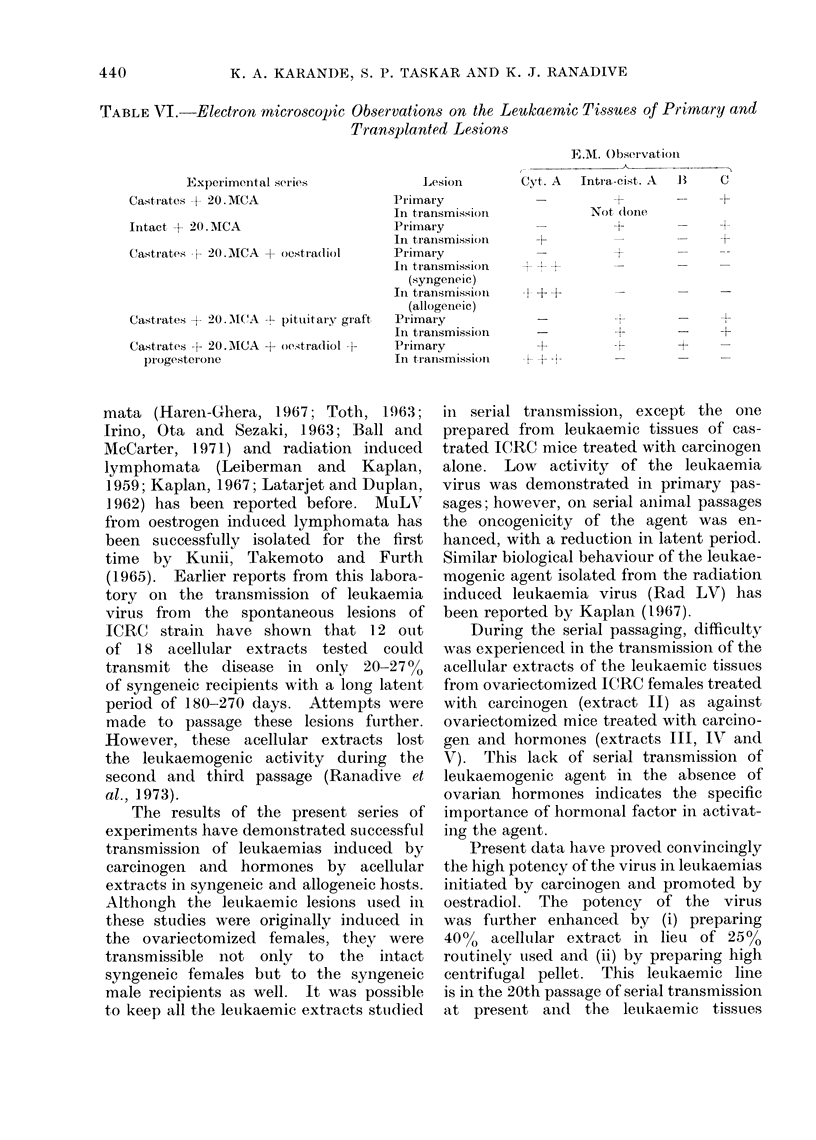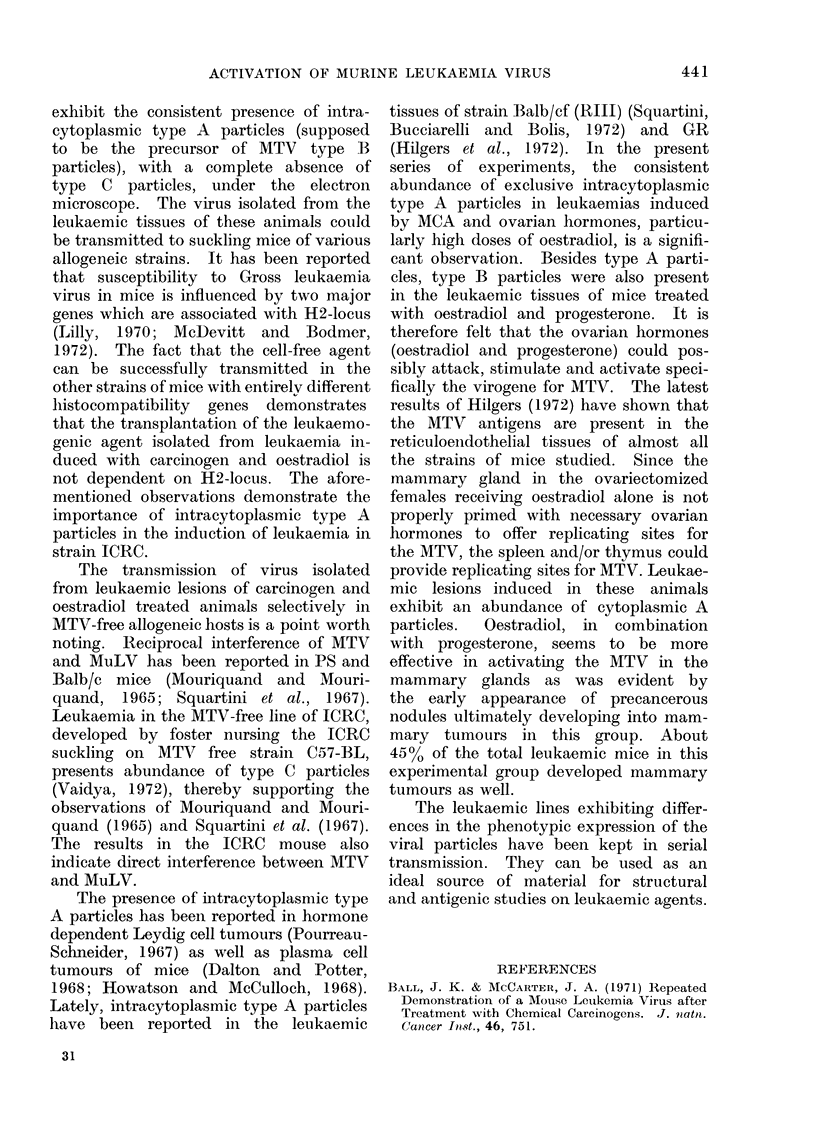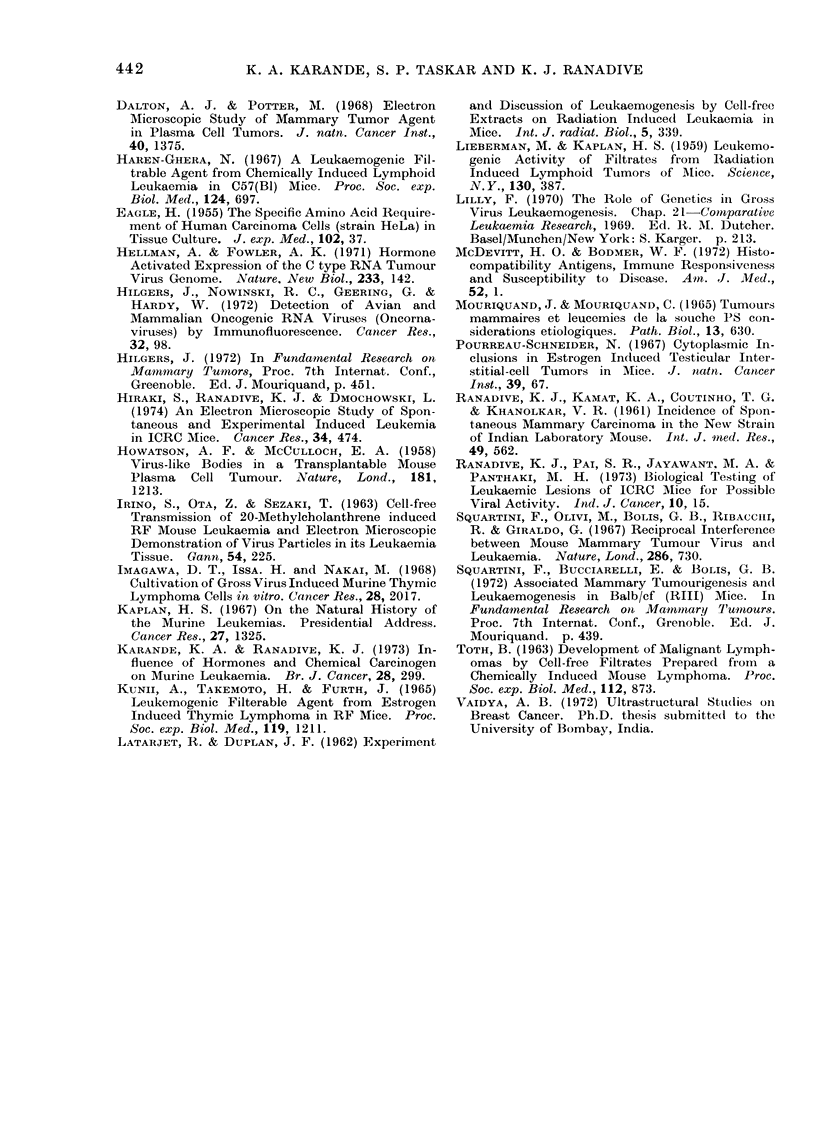# Activation of murine leukaemia virus under different physiological conditions.

**DOI:** 10.1038/bjc.1975.83

**Published:** 1975-04

**Authors:** K. A. Karande, S. P. Taskar, K. J. Ranadive

## Abstract

The leukaemic lesions in intact and ovariectomized mice of strain ICRC, induced with 20-methylcholanthrene (20-MCA) in combination with or without hormones were investigated for the presence of mouse leukaemia virus (MuLV) by (i) bioassays and (ii) electron microscopy. The different experimental groups treated with 20-MCA were (i) intact females, (ii) ovariectomized females, (iii) ovariectomized females with pituitary graft, (iv) ovariectomized females with 10 mug oestradiol/day for 30 days and (v) ovariectomized females with 1 mug oestradiol together with 1 mg progesteron/day for 30 days. It was possible to transmit nearly all these experimentally induced leukaemias to syngeneic mice through acellular extracts, compared with very poor transmissibility of spontaneous leukaemias in the ICRC strain, indicating functional activation of viral agents on combined treatment with carcinogen and hormones. Potency of the acellular leukaemic extract from the mice of group (ii) without the ovarian hormones was much weaker than that from mice of the other experimental groups. The leukaemogenic activity of MuLV was enhanced on serial transmission in syngeneic hosts. Leukaemic lesions of ovariectomized mice treated with 20-MCA and oestradiol were also transmissible to the sucklings of allogeneic mice of strain C3H-MTV, C57-BL and Dba-MTV. The cell-free supernatant medium of the cultures of these leukaemic lesions induced leukaemias on back inoculation into syngeneic mice. Electron microscopic studies of lesions induced with carcinogen and oestradiol consistently showed abundant intracytoplasmic type A particles. Numerous intracytoplasmic type A particles as well as some type B particles were found in the leukaemic tissues of ovariectomized females treated with MCA and oestradiol combined with progesterone. Type C particles, characteristic of MuLV were seen in the leukaemic tissues of all other experimental groups. These findings indicate a significant influence of the physiological condition of the host, particularly the hormonal make up, on expression and activity of specific viral agents.


*Br. J. Cancer* (1975) **31**, 434

### ACTIVATION OF MURINE LEUKAEMIA VIRUS UNDER DIFFERENT PHYSIOLOGICAL CONDITIONS

K. A. KARANDE, S. P. TASKAR AND K. J. RANADIVE

*From the Biology Division, Cancer Research Institute, Tata Memorial Centre, Parel, Bombay* 400 012

Received 23 August 1973. Accepted 9 December 1974

**Summary.—The leukaemic lesions in intact and ovariectomized mice of strain ICRC, induced with 20-methylcholanthrene (20-MCA) in combination with or without hormones were investigated for the presence of mouse leukaemia virus (MuLV) by (i) bioassays and (ii) electron microscopy. The different experimental groups treated with 20-MCA were (i) intact females, (ii) ovariectomized females, (iii) ovariectomized females with pituitary graft, (iv) ovariectomized females with 10 μg oestradiol/day for 30 days and (v) ovariectomized females with 1 μg oestradiol together with 1 mg progesterone/day for 30 days. It was possible to transmit nearly all these experimentally induced leukaemias to syngeneic mice through acellular extracts, compared with very poor transmissibility of spontaneous leukaemias in the ICRC strain, indicating functional activation of viral agents on combined treatment with carcinogen and hormones. Potency of the acellular leukaemic extract from the mice of group (ii) without the ovarian hormones was much weaker than that from mice of the other experimental groups. The leukaemogenic activity of MuLV was enhanced on serial transmission in syngeneic hosts. Leukaemic lesions of ovariectomized mice treated with 20-MCA and oestradiol were also transmissible to the sucklings of allogeneic mice of strain C3H-MTV, C57-BL and Dba-MTV. The cell-free supernatant medium of the cultures of these leukaemic lesions induced leukaemias on back inoculation into syngeneic mice. Electron microscopic studies of lesions induced with carcinogen and oestradiol consistently showed abundant intracytoplasmic type A particles. Numerous intracytoplasmic type A particles as well as some type B particles were found in the leukaemic tissues of ovariectomized females treated with MCA and oestradiol combined with progesterone. Type C particles, characteristic of MuLV were seen in the leukaemic tissues of all other experimental groups. These findings indicate a significant influence of the physiological condition of the host, particularly the hormonal make up, on expression and activity of specific viral agents.**

THE ICRC strain of mice, developed at the Biology Division laboratories of Cancer Research Institute, Bombay is susceptible to the spontaneous development of breast cancer and lymphatic leukaemia. The presence of potent MTV in milk and mammary tissue has been confirmed (Ranadive *et al.*, 1961), and the presence of weak MuLV in spontaneous and transplanted leukaemia of this strain has already been reported (Ranadive *et al.*, 1973). The electron microscopic studies revealed paucity of type C virus particles, characteristic of murine leukaemia, in the spontaneous leukaemic tissues (Hiraki, Ranadive and Dmochowski, 1974). Studies on the experimental induction of leukaemia in castrates of this strain have confirmed the importance of hormones in the acceleration of leukaemogenesis (Karande and Ranadive, 1973). These experimentally induced leukaemic lesions have been investigated for the presence of MuLV by bioassays and electron microscopy. The present communication reports these data evincing activation of the viral agent/s under different physiological conditions.

### MATERIALS AND METHODS

Young virgin ICRC female mice from generations 35–36 were used. For the experimental induction of leukaemia, normal intact and ovariectomized females were given 20-methylcholanthrene (20-MCA) in olive oil, once a week by stomach tube for 8 weeks. The carcinogen treated ovariectomized females were also given a daily dose of oestradiol alone and a dose of oestradiol with progesterone for a period of 30 days. In another group of ovariectomized mice, 2 pituitaries from isologous male mice were grafted subcutaneously onto the right inguinal pair of mammary glands. The detailed experimental procedure has been reported before (Karande and Ranadive, 1973). Extracts of leukaemic lesions were prepared for viral studies from the following groups: Group I (intact virgin mice treated with 20-MCA (Extract I)); Group II (ovariectomized mice treated with 20-MCA (Extract II); Group III (ovariectomized mice with 2 pituitary grafts and treated with 20-MCA (Extract III)); Group IV (ovariectomized mice treated with 20-MCA and 10 $\mu$g oestradiol/day for 30 days (Extract IV)); Group V (ovariectomized mice treated with 20-MCA and 1 $\mu$g oestradiol + 1 mg progesterone/day for 30 days (Extract V)).

*Preparation of acellular extracts of the leukaemic tissues.*—The acellular extracts of the leukaemic tissues were prepared according to Latarjet and Duplan's method (1962). The spleen, mesenteric lymph node and liver were pooled, minced and homogenized in a Teflon homogenizer and suspended in Tyrode's balanced salt solution as either 25% or 40% (w/v) extract. For the initial passages, the leukaemic tissues from the mice with the shortest latent period were used for viral preparation. In the subsequent passages, the transplanted leukaemic lesions from the same passage were pooled together for inoculations. The suspension was centrifuged at 2000 *g* for 20 min at 4°C in the International Centrifuge model HR-1. The supernatant was removed and centrifuged again at 12,000 *g* for 20 min. The upper 2/3rd portion of the clear supernatant fluid was injected intraperitoneally into sucklings (0·2 ml/mouse) or weanlings (0·5 ml/mouse) of syngeneic or allogeneic mice. Since the mortality in the new born mice which received leukaemic extracts was rather high, sucklings more than 4 days old were used for the inoculations. Absence of intact cells from this extract was verified by staining smears of the extract. The allogeneic recipients were from strains ICRC-MTV, Dba-MTV, C3H-MTV, Strong A and C57-BL.

*Preparation of high centrifugal pellet of the leukaemic tissues.*—The leukaemic tissues were minced, homogenized in a Teflon homogenizer and 25% (w/v) extract was prepared in Tyrode's balanced salt solution. The suspension was then centrifuged at 2000 *g* and 12,000 *g* for 20 min each at 4°C. A final centrifugation was done at 98,000 *g* for 90 min. The pellet was then suspended in Tyrode's balanced salt solution and injected intraperitoneally into sucklings of various strains of mice.

In vitro *cultivation of leukaemic lesions.*—The leukaemic lesions were cultivated *in vitro* as static suspension cultures (Imagawa *et al.*, 1968). The medium consisted of BME with a 4 times greater concentration of vitamins and amino acids than usual (Eagle, 1955) supplemented with 15% horse serum. The cultures were incubated at 37°C in an atmosphere of 5% $CO_2$ and 95% air. They were fed with fresh medium on every fourth day, retaining 1 ml of the previous culture medium. Acellular extract, as well as the high centrifugal pellet of the cell free supernatant culture medium were prepared as described above and inoculated into weanlings of syngeneic mice.

*Light and electron microscopy.*—The leukaemic tissues (liver, spleen, thymus, kidneys and lymph nodes) were fixed in Zenker-formol for light microscopy. Tissues for electron microscopy were fixed in 6·25% glutaraldehyde for 1 to $1\frac{1}{2}$ h, post-fixed in 1% osmium tetroxide in Sorensen's buffer, dehydrated in ethanol solutions and embedded in Araldite. The sections were cut on a Porter-5-Blum MT 2 Sorval ultramicrotome, stained in uranyl acetate followed by lead citrate and examined in a Siemens

Elmiskop IA and a RCA EMU G electron microscope*.

RESULTS

The strain ICRC is at present in the 40th generation of inbreeding. The incidence of leukaemia in virgins and breeders of each generation has been studied. The average incidence of leukaemia in the generations F6 to F10 was 42·8%; F11th to F20th, 27·0% and F21st to F30th, 13·9%. The incidence of leukaemia in generations F34–F35, of which the animals have been used for the present study, was 14·6%.

*Cellular transmission of experimentally induced ICRC leukaemia*

All the leukaemic lesions from the experimental groups II, III, IV and V were kept in serial transmission in syngeneic mice without any difficulty, with the exception of those from Group I. Approximately $5 \times 10^7$ cells were injected intraperitoneally in young adult mice. The latent period, which was 1–2 months for the initial 2–3 passages, was reduced to 8 days subsequently.

*Leukaemogenic activity of acellular extracts of lesions induced with 20-MCA in intact and ovariectomized mice*

The leukaemia incidence in intact and ovariectomized ICRC females treated with 20-MCA was 46·1% and 34·4% respectively. About 76% intact females treated with carcinogen, developed mammary cancer as well as leukaemia (Karande and Ranadive, 1973).

On inoculation of extract I (intact virgins treated with 20-MCA) in weanlings of ICRC mice, only one out of 8 animals developed leukaemia in the first passage with a latent period of 160 days (Table I). In further serial passages, the leukaemia incidence increased to 100% with a latent period ranging between 35 and 45 days.

TABLE I.—*Leukaemogenic Activity of Acellular Extracts of 20-MCA Induced Leukaemia in Intact and Ovarietomized ICRC Females*

| Leukaemic extract | Serial passage No. | No. of animals inoculated ♀ | No. of animals inoculated ♂ | No. of animals with leukaemia ♀ | No. of animals with leukaemia ♂ | Latent period (days) |
|---|---|---|---|---|---|---|
| Extract I | 1 | 4 | 4 | — | 1 | 160 |
| | 2 | 2 | 4 | 1 | 4 | 39–50 |
| | 3 | — | 6 | — | 3 | 48–65 |
| | 4 | 6 | 3 | 6 | 3 (100%) | 35–45 |
| | *5 | 5 | 8 | 5 | 8 (100%) | 30–40 |
| Extract II | 1 | 5 | 3 | 1 | 1 | 130–300 |
| | 2 | 4 | 2 | — | — | — |

* The leukaemia is in the 18th passage of serial transmission at present with the latent period of 30–35 days in 100% recipients.

This lesion is at present in its 18th passage of serial transmission. With extract II (ovariectomized females treated with 20-MCA) it was possible to induce leukaemia in 2 out of 8 first passage recipients with a latent period between 130 and 300 days. Further serial transmission was not possible, indicating thereby the low potency of the MuLV. Electron microscopy of the leukaemic tissues of both the primary lesions from which extract I and II were prepared showed few type C particles and intracisternal type A particles (Table VI).

*Leukaemogenic activity of acellular extracts of lesions induced with 20-MCA and pituitary graft*

The data for extract III are shown in Table II. The incidence of leukaemia in ovariectomized ICRC mice treated with 20-MCA along with pituitary graft was 64·7%. The leukaemic lesion used for serial transmission was developed after 3 months of experimental treatment.

In the first passage, 4 animals out of a total number of 17 developed leukaemia

* The electron microscopic studies were carried out by one of the authors (KJR) in collaboration with Drs Hiraki and Dmochowski during her stay as Visiting Professor of Virology at the Department of Virology, M. D. Anderson Hospital and Tumor Institute, University of Texas, Houston, Texas, U.S.A.

TABLE II.—*Serial Transmission of Leukaemia Induced in Ovariectomized ICRC Females with 20-MCA and Pituitary Graft (Extract III)*

| Syngeneic mice | | | | | | Allogeneic mice | | | |
|---|---|---|---|---|---|---|---|---|---|
| Passage no. | No. of animals inoculated ♀ | ♂ | No. of animals c̄ leukaemia ♀ | ♂ (incidence) | Latent period (days) | Material inoculated | Strain and no. of animals inoculated | Age of animals at the time of inoculation (days) | Incidence of leukaemia |
| 1 | 8 | 9 | 1 | 3 | 170, 240, 245, 270 | 40% Acellular extract (passage 3) | ICRC-MTV 8 | 11 | — |
| 2 | 4 | 4 | 2 | 1 | 42, 70, 95 | | | | |
| 3 | 4 | 2 | 4 | 2 (100%) | 40–65 | | Dba-MTV 5 | 10 | — |
| 4 | 3 | 4 | 1 | — | 47 | | | | |
| 5 | 1 | 7 | 1 | 4 (62·5%) | 33–53 | | C57-BL 7 | 9 | — |
| 6 (40%) | 4 | 4 | 1 | — | 52 | | | | |
| 7 (40%) | 5 | 5 | 1 | — (10%) | 55 | | | | |

with a latent period of between 170 and 270 days. The incidence was 100% in the third passage and the latent period was reduced to 40–65 days. From the sixth passage onwards 40% acellular extract was used for inoculation instead of 25%. Even then the incidence of leukaemia was only between 10 and 12% with a latent period of 52–55 days. This line was eventually lost after the 7th serial passage in syngeneic mice. Attempts to transmit this leukaemia in sucklings of allogeneic mice such as ICRC-MTV, Dba-MTV and C57-BL have failed.

The electron microscopic studies carried out on the leukaemic tissues of primary as well as transmitted lesions have demonstrated few or occasional type C particles, invariably accompanied by intracisternal type A particles (Table VI).

TABLE III.—*Serial Transmission of Leukaemia Originally Induced in Ovariectomized ICRC Females with 20-MCA and Oestradiol, in Syngeneic Mice (Extract IV)*

| Passage no. | No. of animals inoculated ♀ | ♂ | No. of animals with leukaemia ♀ | ♂ | Latent period (days) |
|---|---|---|---|---|---|
| 1 | 6 | — | 3 | — | 65, 240, 240 |
| 2 | 7 | 4 | 6 | 4 | 40 |
| 3 | 5 | 3 | 5 | 3 (100%) | 38–40 |
| 4 | 7 | 6 | 6 | 5 (84·6%) | 34–70 |
| 5 | 5 | 3 | 5 | 3 (100%) | 29–60 |
| 6 | 5 | 2 | 2 | 1 (42·8%) | 40–50 |
| 7 | 3 | 3 | — | 1 (16·6%) | 55 |
| *8 (40% extract) | 9 | — | 9 | — (100%) | 34–36 |

* The leukaemia is in the 20th passage of serial transmission at present with the latent period of 30–40 days in 100% recipients.

*Leukaemogenic activity of acellular extracts of lesions induced with carcinogen and oestradiol*

The data on extract IV are tabulated in Tables III and IV. The incidence of leukaemia in ovariectomized ICRC females treated with 20-MCA and oestradiol was 81·2%. About 12·5% females developed mammary cancer. One female developed mammary cancer as well as leukaemia.

On inoculation of the leukaemic extract in syngeneic mice, the incidence of leukaemia in the first passage was 50%, with the latent period ranging between

TABLE IV.—*Serial Transmission of Leukaemia Originally Induced in Ovariectomized ICRC Females with 20-MCA and Oestradiol, in Allogeneic Mice (Extract IV)*

| Leukaemic extract | Material inoculated | Total no. of animals inoculated | Strain and no. of animals inoculated | Age of animals at the time of inoculation (days) | Incidence of leukaemia | Latent period (days) |
|---|---|---|---|---|---|---|
| Extract IV (Passage 4) | Acellular extract (40% homogenate) | 18 | ICRC-MTV 5 | 6 | 3/5 | 43–45 |
| | | | Dba-MTV 3 | 7 | 2/3 | 45, 85 |
| | | | Strong A 3 | 7 | — | — |
| | | | C3H Jax 2 | 6 | — | — |
| | | | *C3H-MTV 5 | 8 | 1/5 | 36 |
| Extract IV | High centrifugal pellet | 36 | ICRC 5 | 21 | 3/5 | 45, 51 66 |
| | | | ICRC-MTV 12 — 6 | 5 | 3/6 | 44 |
| | | | 6 | 14 | — | — |
| | | | †Dba-MTV 5 | 6 | 3/5 | 111, 150 150 |
| | | | Strong A 6 | 8 | — | — |
| | | | ‡C57-BL 6 | 8 | 1/6 | 82 |
| | | | C3H-Jax 2 | 4 | — | — |

* The line is in 2nd passage of transmission in syngeneic mice.
† The line is in 4th passage of transmission in syngeneic mice.
‡ The line is in 4th passage of transmission in syngeneic mice.

65 and 240 days. By the third passage, all the inoculated mice developed leukaemia, the latent period being reduced to 38–40 days. As there was a gradual decrease in the leukaemia incidence in the subsequent passages, 40% acellular extract instead of 25% was used for further inoculations. This resulted in increased incidence (100%) with a latent period of 34–36 days. This leukaemic lesion designated as "IE2" has been consistently transmissible through acellular extracts and is at present in its 20th passage of serial transmission.

*Transmission of leukaemia in allogeneic mice*

The data have been presented in Table IV. Leukaemic tissues from a batch of animals in the same passage were pooled and either 40% acellular extract or high centrifugal pellet was prepared. The acellular MuLV preparations were inoculated into the sucklings of allogeneic strains. It was possible to transmit the leukaemia in sucklings of allogeneic hosts such as ICRC-MTV, Dba-MTV, C57-BL and C3H-MTV, the strains devoid of mammary tumour virus. After inoculation of 40% acellular extract of the leukaemic tissues, 2 out of 3 Dba-MTV animals, 3 out of 5 ICRC-MTV animals and one out of 5 C3H-MTV animals developed leukaemia with the latent period comparable with that in the parent line. The inoculations were done at the ages between 6 and 8 days. Strains Strong A and C3H(Jax) having potent MTV have not yet developed leukaemia, the animals at present being 355 and 270 days old respectively.

With the inoculation of high centrifugal pellet of the leukaemic tissue extract, it was possible to transmit the leukaemia in strain C57-BL suckling mice. One out of a total of 6 inoculated mice developed leukaemia with a latent period of 82 days. In strain ICRC-MTV, one out of 6 inoculated mice developed leukaemia when the animals were injected at the age of 5 days. The group of ICRC-MTV animals, inoculated at the age of 14 days, did not develop leukaemia, indicating the importance of age at the time of inoculation in the development of the disease. Attempts were also made to transmit the induced lesions of strains Dba-MTV, C3H-MTV and C57BL in the respective syngeneic mice (Table IV). At present the leukaemic lesions are in 4th, 2nd and 4th serial passages of transmission in strain Dba-MTV, C3H-MTV and C57-BL respectively.

Electron microscopic studies carried out on the tissues of primary leukaemia lesions as well as subsequent passages of strain ICRC mice revealed the absence of type C particles characteristic of murine leukaemia virus. On the other hand, almost all the tissues studied presented an abundance of intracytoplasmic type A virus particles consistently. The E.M. studies carried out on some of the leukaemic lesions of strains Dba-MTV and C57-BL also showed an abundance of intracytoplasmic A particles similar to those observed in the parent line.

### In vitro *studies*

Three out of 5 animals inoculated with high centrifugal pellet of the cell-free supernatant culture medium of leukaemic spleens of this IE2 line developed leukaemia within the latent period of 2–7 months.

### *Leukaemogenic activity of acellular extracts of lesions induced with carcinogen together with oestradiol and progesterone*

The data on extract V are tabulated in Table V. The incidence of leukaemia in ovariectomized ICRC females treated with 20-MCA along with oestradiol and progesterone was 73·3%. The mammary cancer incidence in this group was 40%.

TABLE V.—*Serial Transmission of Leukaemia Induced in Ovariectomized ICRC Females with 20-MCA along with Oestradiol and Progesterone (Extract V)*

| Passage no. | No. of animals inoculated ♀ | No. of animals inoculated ♂ | No. of animals with leukaemia ♀ | No. of animals with leukaemia ♂ | Latent period |
|---|---|---|---|---|---|
| 1 | 6 | 8 | 4 | 3 | 180–240 |
| | | | (50%) | | |
| 2 | 5 | 5 | 3 | 2 | 90–120 |
| | | | (50%) | | |
| 3 | 4 | 6 | 1 | 1 | 90 |
| | | | (20%) | | |

About 45% leukaemic females also developed mammary cancer.

On inoculation of acellular extract of these leukaemic lesions, 50% syngeneic recipients of the first passage developed leukaemia with the latent period ranging between 180 and 240 days. In the third passage, about 20% animals developed leukaemia with an average latent period of 90 days. This line was ultimately lost after the 4th serial passage. Electron microscopic studies carried out on some of the leukaemia lesions showed an abundance of intracytoplasmic A particles with complete absence of type C particles. Type B particles were occasionally observed in some of the leukaemic spleens.

### DISCUSSION

The role of hormones in accelerating leukaemogenesis in strain ICRC has been studied previously (Karande and Ranadive, 1973). Treatment with chemical carcinogens accelerated the process of leukaemogenesis in castrated ICRC mice, particularly when the carcinogen was administered together with the hormones. This demonstrated the importance of the physiological environment of the host in controlling oncogene activation. The possibility that the hormonal status during the life span of the host is influential in both the repression and derepression of the oncogene has been suggested recently by Hellman and Fowler (1971).

The successful isolation of a filterable MuLV from chemically induced lympho-

TABLE VI.—*Electron microscopic Observations on the Leukaemic Tissues of Primary and Transplanted Lesions*

| Experimental series | Lesion | E.M. Observation Cyt. A | Intra-cist. A | B | C |
|---|---|---|---|---|---|
| Castrates + 20.MCA | Primary | — | + | — | + |
| | In transmission | | Not done | | |
| Intact + 20.MCA | Primary | — | + | — | + |
| | In transmission | + | — | — | + |
| Castrates + 20.MCA + oestradiol | Primary | — | + | — | — |
| | In transmission (syngeneic) | +++ | — | — | — |
| | In transmission (allogeneic) | +++ | — | — | — |
| Castrates + 20.MCA + pituitary graft | Primary | — | + | — | + |
| | In transmission | — | + | — | + |
| Castrates + 20.MCA + oestradiol + progesterone | Primary | + | + | + | — |
| | In transmission | +++ | — | — | — |

mata (Haren-Ghera, 1967; Toth, 1963; Irino, Ota and Sezaki, 1963; Ball and McCarter, 1971) and radiation induced lymphomata (Leiberman and Kaplan, 1959; Kaplan, 1967; Latarjet and Duplan, 1962) has been reported before. MuLV from oestrogen induced lymphomata has been successfully isolated for the first time by Kunii, Takemoto and Furth (1965). Earlier reports from this laboratory on the transmission of leukaemia virus from the spontaneous lesions of ICRC strain have shown that 12 out of 18 acellular extracts tested could transmit the disease in only 20–27% of syngeneic recipients with a long latent period of 180–270 days. Attempts were made to passage these lesions further. However, these acellular extracts lost the leukaemogenic activity during the second and third passage (Ranadive *et al.*, 1973).

The results of the present series of experiments have demonstrated successful transmission of leukaemias induced by carcinogen and hormones by acellular extracts in syngeneic and allogeneic hosts. Althongh the leukaemic lesions used in these studies were originally induced in the ovariectomized females, they were transmissible not only to the intact syngeneic females but to the syngeneic male recipients as well. It was possible to keep all the leukaemic extracts studied in serial transmission, except the one prepared from leukaemic tissues of castrated ICRC mice treated with carcinogen alone. Low activity of the leukaemia virus was demonstrated in primary passages; however, on serial animal passages the oncogenicity of the agent was enhanced, with a reduction in latent period. Similar biological behaviour of the leukaemogenic agent isolated from the radiation induced leukaemia virus (Rad LV) has been reported by Kaplan (1967).

During the serial passaging, difficulty was experienced in the transmission of the acellular extracts of the leukaemic tissues from ovariectomized ICRC females treated with carcinogen (extract II) as against ovariectomized mice treated with carcinogen and hormones (extracts III, IV and V). This lack of serial transmission of leukaemogenic agent in the absence of ovarian hormones indicates the specific importance of hormonal factor in activating the agent.

Present data have proved convincingly the high potency of the virus in leukaemias initiated by carcinogen and promoted by oestradiol. The potency of the virus was further enhanced by (i) preparing 40% acellular extract in lieu of 25% routinely used and (ii) by preparing high centrifugal pellet. This leukaemic line is in the 20th passage of serial transmission at present and the leukaemic tissues exhibit the consistent presence of intracytoplasmic type A particles (supposed to be the precursor of MTV type B particles), with a complete absence of type C particles, under the electron microscope. The virus isolated from the leukaemic tissues of these animals could be transmitted to suckling mice of various allogeneic strains. It has been reported that susceptibility to Gross leukaemia virus in mice is influenced by two major genes which are associated with H2-locus (Lilly, 1970; McDevitt and Bodmer, 1972). The fact that the cell-free agent can be successfully transmitted in the other strains of mice with entirely different histocompatibility genes demonstrates that the transplantation of the leukaemogenic agent isolated from leukaemia induced with carcinogen and oestradiol is not dependent on H2-locus. The aforementioned observations demonstrate the importance of intracytoplasmic type A particles in the induction of leukaemia in strain ICRC.

The transmission of virus isolated from leukaemic lesions of carcinogen and oestradiol treated animals selectively in MTV-free allogeneic hosts is a point worth noting. Reciprocal interference of MTV and MuLV has been reported in PS and Balb/c mice (Mouriquand and Mouriquand, 1965; Squartini *et al.*, 1967). Leukaemia in the MTV-free line of ICRC, developed by foster nursing the ICRC suckling on MTV free strain C57-BL, presents abundance of type C particles (Vaidya, 1972), thereby supporting the observations of Mouriquand and Mouriquand (1965) and Squartini *et al.* (1967). The results in the ICRC mouse also indicate direct interference between MTV and MuLV.

The presence of intracytoplasmic type A particles has been reported in hormone dependent Leydig cell tumours (Pourreau-Schneider, 1967) as well as plasma cell tumours of mice (Dalton and Potter, 1968; Howatson and McCulloch, 1968). Lately, intracytoplasmic type A particles have been reported in the leukaemic tissues of strain Balb/cf (RIII) (Squartini, Bucciarelli and Bolis, 1972) and GR (Hilgers *et al.*, 1972). In the present series of experiments, the consistent abundance of exclusive intracytoplasmic type A particles in leukaemias induced by MCA and ovarian hormones, particularly high doses of oestradiol, is a significant observation. Besides type A particles, type B particles were also present in the leukaemic tissues of mice treated with oestradiol and progesterone. It is therefore felt that the ovarian hormones (oestradiol and progesterone) could possibly attack, stimulate and activate specifically the virogene for MTV. The latest results of Hilgers (1972) have shown that the MTV antigens are present in the reticuloendothelial tissues of almost all the strains of mice studied. Since the mammary gland in the ovariectomized females receiving oestradiol alone is not properly primed with necessary ovarian hormones to offer replicating sites for the MTV, the spleen and/or thymus could provide replicating sites for MTV. Leukaemic lesions induced in these animals exhibit an abundance of cytoplasmic A particles. Oestradiol, in combination with progesterone, seems to be more effective in activating the MTV in the mammary glands as was evident by the early appearance of precancerous nodules ultimately developing into mammary tumours in this group. About 45% of the total leukaemic mice in this experimental group developed mammary tumours as well.

The leukaemic lines exhibiting differences in the phenotypic expression of the viral particles have been kept in serial transmission. They can be used as an ideal source of material for structural and antigenic studies on leukaemic agents.

REFERENCES

Ball, J. K. & McCarter, J. A. (1971) Repeated Demonstration of a Mouse Leukemia Virus after Treatment with Chemical Carcinogens. *J. natn. Cancer Inst.*, **46**, 751.

DALTON, A. J. & POTTER, M. (1968) Electron Microscopic Study of Mammary Tumor Agent in Plasma Cell Tumors. *J. natn. Cancer Inst.*, **40**, 1375.

HAREN-GHERA, N. (1967) A Leukaemogenic Filtrable Agent from Chemically Induced Lymphoid Leukaemia in C57(Bl) Mice. *Proc. Soc. exp. Biol. Med.*, **124**, 697.

EAGLE, H. (1955) The Specific Amino Acid Requirement of Human Carcinoma Cells (strain HeLa) in Tissue Culture. *J. exp. Med.*, **102**, 37.

HELLMAN, A. & FOWLER, A. K. (1971) Hormone Activated Expression of the C type RNA Tumour Virus Genome. *Nature, New Biol.*, **233**, 142.

HILGERS, J., NOWINSKI, R. C., GEERING, G. & HARDY, W. (1972) Detection of Avian and Mammalian Oncogenic RNA Viruses (Oncornaviruses) by Immunofluorescence. *Cancer Res.*, **32**, 98.

HILGERS, J. (1972) In *Fundamental Research on Mammary Tumors*, Proc. 7th Internat. Conf., Greenoble. Ed. J. Mouriquand, p. 451.

HIRAKI, S., RANADIVE, K. J. & DMOCHOWSKI, L. (1974) An Electron Microscopic Study of Spontaneous and Experimental Induced Leukemia in ICRC Mice. *Cancer Res.*, **34**, 474.

HOWATSON, A. F. & MCCULLOCH, E. A. (1958) Virus-like Bodies in a Transplantable Mouse Plasma Cell Tumour. *Nature, Lond.*, **181**, 1213.

IRINO, S., OTA, Z. & SEZAKI, T. (1963) Cell-free Transmission of 20-Methylcholanthrene induced RF Mouse Leukaemia and Electron Microscopic Demonstration of Virus Particles in its Leukaemia Tissue. *Gann*, **54**, 225.

IMAGAWA, D. T., ISSA. H. and NAKAI, M. (1968) Cultivation of Gross Virus Induced Murine Thymic Lymphoma Cells *in vitro*. *Cancer Res.*, **28**, 2017.

KAPLAN, H. S. (1967) On the Natural History of the Murine Leukemias. Presidential Address. *Cancer Res.*, **27**, 1325.

KARANDE, K. A. & RANADIVE, K. J. (1973) Influence of Hormones and Chemical Carcinogen on Murine Leukaemia. *Br. J. Cancer*, **28**, 299.

KUNII, A., TAKEMOTO, H. & FURTH, J. (1965) Leukemogenic Filterable Agent from Estrogen Induced Thymic Lymphoma in RF Mice. *Proc. Soc. exp. Biol. Med.*, **119**, 1211.

LATARJET, R. & DUPLAN, J. F. (1962) Experiment and Discussion of Leukaemogenesis by Cell-free Extracts on Radiation Induced Leukaemia in Mice. *Int. J. radiat. Biol.*, **5**, 339.

LIEBERMAN, M. & KAPLAN, H. S. (1959) Leukemogenic Activity of Filtrates from Radiation Induced Lymphoid Tumors of Mice. *Science, N.Y.*, **130**, 387.

LILLY, F. (1970) The Role of Genetics in Gross Virus Leukaemogenesis. Chap. 21—*Comparative Leukaemia Research*, 1969. Ed. R. M. Dutcher. Basel/Munchen/New York: S. Karger. p. 213.

MCDEVITT, H. O. & BODMER, W. F. (1972) Histocompatibility Antigens, Immune Responsiveness and Susceptibility to Disease. *Am. J. Med.*, **52**, 1.

MOURIQUAND, J. & MOURIQUAND, C. (1965) Tumours mammaires et leucemies de la souche PS considerations etiologiques. *Path. Biol.*, **13**, 630.

POURREAU-SCHNEIDER, N. (1967) Cytoplasmic Inclusions in Estrogen Induced Testicular Interstitial-cell Tumors in Mice. *J. natn. Cancer Inst.*, **39**, 67.

RANADIVE, K. J., KAMAT, K. A., COUTINHO, T. G. & KHANOLKAR, V. R. (1961) Incidence of Spontaneous Mammary Carcinoma in the New Strain of Indian Laboratory Mouse. *Int. J. med. Res.*, **49**, 562.

RANADIVE, K. J., PAI, S. R., JAYAWANT, M. A. & PANTHAKI, M. H. (1973) Biological Testing of Leukaemic Lesions of ICRC Mice for Possible Viral Activity. *Ind. J. Cancer*, **10**, 15.

SQUARTINI, F., OLIVI, M., BOLIS, G. B., RIBACCHI, R. & GIRALDO, G. (1967) Reciprocal Interference between Mouse Mammary Tumour Virus and Leukaemia. *Nature, Lond.*, **286**, 730.

SQUARTINI, F., BUCCIARELLI, E. & BOLIS, G. B. (1972) Associated Mammary Tumourigenesis and Leukaemogenesis in Balb/cf (RIII) Mice. In *Fundamental Research on Mammary Tumours*. Proc. 7th Internat. Conf., Grenoble. Ed. J. Mouriquand. p. 439.

TOTH, B. (1963) Development of Malignant Lymphomas by Cell-free Filtrates Prepared from a Chemically Induced Mouse Lymphoma. *Proc. Soc. exp. Biol. Med.*, **112**, 873.

VAIDYA, A. B. (1972) Ultrastructural Studies on Breast Cancer. Ph.D. thesis submitted to the University of Bombay, India.